# Rehabilitation of patients with peripheral arterial disease

**DOI:** 10.1016/j.amsu.2021.102864

**Published:** 2021-09-14

**Authors:** M. Noumairi, A. Bouallala, S. EL Mir, A. Allam, A.A. EL Oumri

**Affiliations:** a Department of Physical Medicine and Rehabilitation,university Hospital, MOHAMMED VI OUJDA, Morocco; b Department of Vascular Surgery,university Hospital, MOHAMMED VI OUJDA Morocco; c Department of Physical Medicine Rheumatology and Rehabilitation Faculty of Medicine Tanta University, Egypt

**Keywords:** Vascular rehabilitation, PAOD, Walking perimeter

## Abstract

Vascular rehabilitation is an essential and effective treatment of peripheral arterial obstructive disease (PAOD). It is recommended in the first line by the European and American scientific societies**.** The rehabilitation to the walk remains the basic treatment of the arteriopathy of the lower limbs. Different walking protocols can be proposed. For similar effectiveness, vascular rehabilitation consists of an outpatient or specialized institution management program based on a comprehensive approach involving all or many of the following techniques: relaxation, active analytical exercises, gait rehabilitation, exercise re-entry, physical activities adapted to the patient's daily life and illness, respiratory physiotherapy, therapeutic education of the patient, smoking cessation aid.

Vascular rehabilitation of arteritis requires a variety of skills but still faces a clear lack of suitable structures; it remains poorly prescribed and poorly known by usual prescribers (general practitioner, vascular surgeon)**.**

## Highlights

• Vascular rehabilitation is the first-line treatment of stress ischemia in patients with peripheral arterial obstructive disease (PAOD).
• It allows to significantly increase the maximum walking distance.
• Its prescription must be integrated into the management networks of cardiovascular diseases.

## 1 Introduction

Many scientific societies recommend first-line vascular rehabilitation (Level A) for patients with intermittent claudication (Stage 2 of Leriche and Fontaine classification) [1,2], it presents a daily challenge for many reasons; to obtain the patient's membership in the exercise program, integrate the arteriolopath into a management that must be multidisciplinary because of the multifactorial character of the disease and know how to adapt the protocol according to the severity of the disease and its extension to other territories [3]. Gait rehabilitation remains the basic treatment for lower limb arteropathy. Different walking protocols can be proposed, the orientation of any patient with an effort ischemia to a rehabilitation center for walking should be systematic like cardiac rehabilitation [4].

### 1.1 Epidemiology

PAOD is a common systemic disease, especially at stage 1 and stage 2 of the LERICH and FONTAINE classification. The patient with PAOD has a poly arterial disease that can affect other areas including the heart and brain. In the international register REACH, half of the patients with PAOD had at least one other arterial territory affected against 1/3 of cerebrovascular patients and 1/4 of patients with coronary artery disease [5,6].

Indeed, a claudication questionnaire was used in European and American studies which objected that the prevalence of intermittent claudication was 1% before the age of 50 and reached only 6% after the age of 65. The prevalence of PAOD also increased by almost 25% between 2000 and 2010**,** its frequency was of the order of 5% by the age of 50, then increases in both men and women to reach a frequency of the order of 20% after 80 years in high-income countries [6,7].

The main vascular risk factors that promote the development of PAOD are: smoking and diabetes**,** the first double or triple the risk of PAOD, and the second double or quadruple with an increase in the risk related to the seniority of diabetes. High blood pressure, high cholesterol in LDL are also associated with PAOD but less so**.** [6].

### 1.2 Pathophysiology

With aging, all sclerosis including the arteries. This inevitable evolution is pathogenic. With age, the hardening of the artery wall extends. This arteriosclerosis transforms the arterial tree into a rigid “pipe ”, less and less able to change its caliber and regulate blood flow. Little by little, the periphery is less well fed [[8], [9], [10], [11]], this process is accelerated by the presence of other cardiovascular risk factors (diabetes, hypertension, obesity, tobacco, male gender, female after menopause, etc.) Therefore one will have a formation of a plaque of atherosm that obstructs as the arterial light decreases blood flow imbalance between the intakes and clinical symptomatology needs (intermittent claudication) [10,11].

• Mechanisms for improving walking distance include:

### 1.3 Metabolic changes

PAOD causes mitochondrial dysfunction by accumulation of intermediate metabolites (acylcarnitin).

Regular gait training reduces the concentration of acylcarnitin and improves mitochondrial function [12].

### 1.4 Stimulation of angiogenesis

Physical exercise induces an increase in VEGF and HIF-1 (factors induced by hypoxia), which promote angiogenesis [12].

### 1.5 Improvement of biomechanical factors

It has been shown that the trained patient will decrease the muscle oxygen consumption by better

Support of the movement and the walking pattern [12].

### 1.6 Haemorrhagic factors

The blood viscosity and plasticity of red blood cells are improved by exercise [12].

### 1.7 Inflammatory factors

Classically raised in the PAOD and markers of a pejorative cardiovascular evolution, they decrease after 3 months of rehabilitation [12].

### 1.8 Endothelial factors

Several studies have shown an improvement in endothelial function after re-training.

The importance of this function has been highlighted for its role in improving walking distance in patients working only the upper limbs [12].

### 1.9 Improvement of the pain threshold

Found in a study by Gardner, it allows a better adhesion of the patient [12].

### 1.10 Management of risk factors

Exercise improves blood pressure profile, lowers LDL cholesterol, regulates diabetes and helps stabilize body weight [12].

### 1.11 Improvement of mood disorders

With increased activities of daily living, decreased reactive depressive syndrome, improved cognitive function [12].

### 1.12 Walking rehabilitation techniques

Vascular rehabilitation is currently recommended in first line (level A) in patients with intermittent claudication - ischemia of effort-by many scientific societies for many reasons, first, the impact on quality of life through the improvement of self-esteem inherent in the improvement of functional abilities, and then the action on morbidity-mortality through participation in a therapeutic education programme [13,14]. This program is essential to the maintenance of long-term hygienodietetic rules taking into account smoking cessation, therapeutic adherence (statins, antiplatelet agents, inhibitors of the conversion enzyme), adapted and regular physical activity and nutrition education of course, the duration, frequency and intensity of physical activity should be prescribed according to specific recommendations, and finally the improvement of walking distance which allows an increase in walking distance by an average of 150% which would be explained by the improvement of extraction and peripheral use of oxygen [[14], [15], [16]].

The main mechanisms involved in improving walking distance include cardiorespiratory, circulatory, biochemical adaptations (action of growth factors such as VEGF and FGF-2) and musculoskeletal as well synthesized by Brenner et al., in a recent study [17].

The rehabilitation can be done in a rehabilitation centre specialized in vascular pathologies in the context of a complete hospitalization or ambulatory; it can also be done at home, Some authors have also shown a substantially identical effectiveness over walking distance, in patients re-educated in centres versus in homes under supervision [18,19] Regular walking activity should be offered to all patients with PAOD [19].

### 1.13 Indications for gait rehabilitation are currently

- Effort ischemia, especially for femoral lesions, patients with iliac or distal lesions also but with less satisfactory results [20].
- Resting ischemia in a patient with surgical contraindication. In this case, it is preferable to carry it out in a specialized centre [20].
- Following surgery or endovascular techniques or even amputation. This therapeutic orientation should be considered in conjunction with the vascular surgeon [20].

The contraindications of vascular rehabilitation are the presence of an unstable cardiac, pulmonary or cerebral pathology. That is, any contra-indication to effort [21]. It is necessary to carry out an initial assessment which must be global and include careful examination and clinical examination with palpation of the peripheral pulses and auscultation of vascular routes [22]. Arterial assessment of the lower limbs (Doppler echo, IPSc) and if possible microcirculatory (TcPO2), detect other locations of atheromatous disease such as ischemic heart disease, carotid stenosis or abdominal aortic aneurysm. The assessment is complemented by a systematic search for an impairment of the musculoskeletal system that may require an adaptation of the rehabilitation program [22].

For many years, scientific societies have advocated the “Gardner protocol” [19] of walking 30–60 min at least 3 times a week for 3–6 months. If possible, claudication should occur in less than 10 min, In this protocol, the patient should continue to walk after the appearance of the first discomfort until the occurrence of a so-called moderate pain assessed at 7/10 on the visual analogue scale (VAS), stop and especially do not continue to walk from this stage reached, Observe a rest period of at least 5 min before resuming walking [19].

The proposed protocol must of course adapt to each patient characteristics and any associated diseases. Walking rehabilitation with the onset of pain at each walking episode leads to better results, but may discourage fragile patients who do not want to suffer. Gardner A.W. has shown that, at equivalent work volume, a lower intensity of effort (40% versus 80%) allows getting identical results [23].

The training interval method consists of a split-type workout alternating a period of high intensity work and an active recovery period with sub-maximum intensity effort. This method is used in cardiology with good results. In 2011, Villemur B. et al., in a preliminary study, tested this protocol in stress ischemia and showed a doubling of walking distance in 2 weeks. This result requires confirmation [24].

The required duration of the support is indeterminable as the factors are numerous and variable and so the anomalies persist and grow. A consensus nevertheless recommends an initial program of supervised exercises [25] consisting of at least two [26] to three [27] weekly sessions of 30–60 min. The length of sessions is usually adapted to the possibilities. It is 30 min for sessions twice daily and 60 min for monodaily [28]. The program is conducted on 1 [29], 3 [28,[30], [31], [32]], 4 [33], 6 months [[33], [34], [35]] or 12 [27] to 15 months [26]. The many restrictions or adaptations result from the multifocal nature of the disease, the presence of associated disabilities [30,36] and special circumstances (amputation, hemiplegia, obesity) [28,37,38] The variety of rehabilitation programs is considerable.

A vascular rehabilitation programme consists, at a minimum, of three weekly 1-h sessions for a period of three to six months in a specialized centre, under supervision at home or in a physiotherapy practice [39]. In all cases, the programme must have two complementary axes: reconditioning with effort and therapeutic education of the patient. There are two phases to reconditioning to effort. The evaluation phase followed by the re-entrainment phase. The purpose of the evaluation is to quantify the limitation to the patient's walking, and to guide the modalities of the re-entrainment by assessing the ability to adapt to the effort and detect coronary intolerance or other contra-indication [39,40]. Several methods of assessing capacity for physical activity are possible:

- Measurement of walking distance on flat ground until the onset of cramp: poorly standardized test and low reproducibility [39]. This step nevertheless makes it possible to look for a deformation of the step that can be found in the severe claudicant and which will require a correction if necessary
- 6-min walking test: standardized and validated in the assessment of chronic disabilities. Explore enduring capabilities with good reproducibility [40].
- Treadmill tests to monitor patient progress under standardised and therefore reproducible speed and slope conditions. Most often these are tests with a gradual increase in work [41]. Treadmill tests can be constant load (fixed speed and slope) or progressive [42].
- Ergometric bicycle test: May be useful in case of obesity or walking disorders related to orthopedic or neurological impairments. This test is interesting in respiratory and cardiology, but does not test the handicap related to arterial disease
- Upper limb ergometer stress test: Is a good alternative when lower limbs are not not useable with a sensitivity close to that of a treadmill test [43].

The second phase of retraining is personalized, adapted to each case and takes into account the results obtained by the patient during the evaluation phase. The protocol put in place may evolve according to the evolution of arterial disease. It is important to set appropriate and realistic goals so as not to discourage patients [40].

There are two main types of techniques that are often associated:

The overall training and analytical reconditioning.The overall training consists of performing various walking exercises either on a treadmill or on a cycloergometer in order to solicit the lower limbs, the upper limbs and thus recruit a maximum of muscle volume. Ergometric reconditioning involving the upper limbs and trunk may be useful when the lower limbs are not useable with a positive systemic effect, improving tissue oxygenation at lower limb level [44].

The analytical (segmental) reconditioning of the lower limbs is the set of techniques that target muscle building by muscle contraction against progressive resistance as well as increased blood flow in the arterial network. It is less often advocated because systemic effects are less important compared to overall training [45]. Dynamic muscle contractions at a frequency of 20–50 contractions per minute against progressive resistance and according to clinical tolerance are achieved by avoiding the occurrence of muscle cramp.

Other rehabilitation techniques can be applied according to situations such as respiratory physiotherapy in case of associated COPD, lymphatic drainage in case of edema, mobilization in case of retractions and stiffness, postural rehabilitation in case of balance disorders, such as limb swaying and proprioception exercises, so-called reflex massages to stimulate microcirculation, interesting in case of trophic disorder or very close claudication, or walking in an aquatic environment when the walking perimeter is very limited. Finally, credo therapy, which is part of the physical therapeutic arsenal, can be proposed [44,45].

### 1.14 Flexibilities

The work of relaxation or stretching is an essential prerequisite for muscular and endurance work. It also improves flexibility [44,45].

### 1.15 Active analytical exercises

Segmental muscle building improves the strength of the muscles and functional capacity, the strength of the contractions is fixed at 20–50% of the maximum voluntary force previously evaluated so as to stay on aerobic work [44,45].

### 1.16 Rehabilitation to walking

Walking exercises are daily and based on the initial assessment of the perimeter of embarrassment. The walking distance worked is fixed at 80% of the perimeter of discomfort, as well as the recovery time which is fixed at 80% of the time of disappearance of the pain [44,45].

### 1.17 Work conditioning

Sessions last from 30 to 45 min. The intensity of re-training is always based on the training heart rate and the dyspnea threshold. The sessions include a 30-min endurance phase at the target heart rate, preceded by a warm-up phase and followed by an active recovery phase. The endurance phase can be maintained in a constant load plateau or by alternating periods of lower intensity and peaks of activity [44,45].

The effort re-training can be done on different ergometers (bicycle, hand ergometer, treadmill, stepper, rowing machine) but can also be an outdoor walk, outdoor bike, swimming … [44,45].

### 1.18 Respiratory physiotherapy

The work of ventilatory control is interesting, throughout the rehabilitation, in patients often « heavy smokers».It allows the awareness of the abdominodiaphragmatic breathing and thus improve the intake of O2 [44,45].

### 1.19 Therapeutic education of the patient

Therapeutic education is an integral part of the care program. It should allow the sustainability of the results achieved during the program.

The main topics covered are treatment, dyslipidemia, excess weight, blood pressure control, diabetes, tobacco, physical activity, foot care [44,45].

### 1.20 Outcomes of rehabilitation protocols

Gardner's meta-analysis shows a 180% increase in the distance of onset of pain with a result from the first 2 months. Watson et al. [16].

In a 2008 Cochrane review, which included 22 randomized studies, walking distance improved by more than medical treatment and by 200% with no cardiovascular complications and maintained over 2 years [46].

In rehabilitation centres, with a higher volume of work, the walking distance can be increased from 4 to 5 times. Femoral lesions perform best, but iliac and distal lesions can also improve significantly. The lameness of buttocks is more moderate. Fokkenrood [46] shows in a 2013 Cochrane review superior results in patients who received rehabilitation in the centre, compared to the home with a distance gain of 160 m and a result maintained at 1 year. Supervision improves patient adherence and exercise intensity. It is very recommended by scientific societies because of its favourable cost-effectiveness ratio [47].

The recent ERASE study compared the increase in maximum walking distance in patients with claudication related to iliac or femoral artery injury treated either by endovascular treatment alone or by the combination of endovascular treatment and gait rehabilitation Gardner protocol. The combination of endovascular treatment and walking rehabilitation resulted in a better result at one year with a maximum walking distance multiplied by 5.7. In the walk-alone rehabilitation group, maximum walking distance was also significantly improved (WMD 4.35) [47] The 2013 Cochrane review showed better outcomes for patients who received rehabilitation in structured centre compared to those who received self-reported home care: in the centre, the additional distance gain was 160 m with the result maintained at 1 year [48].

## 2 Conclusion

Vascular rehabilitation is the first-line treatment of stress ischemia in patients with PAOD. It allows to significantly increase the maximum walking distance especially in case of femoral injuries, it also allows to improve the balance of risk factors, cognitive functions and activities of daily life. It is just as effective as endovascular treatment for long-term femoral lesions. It should be carried out preferentially in a supervised manner but can be prescribed as an outpatient with minimal supervision, it must be adapted and multidisciplinary. It corresponds to an obvious need but not satisfied by the manifest lack of suitable structures. It remains poorly prescribed and poorly known to usual prescribers (cardiologist, vascular surgeons, general practitioners). Its prescription must be integrated into the management networks of cardiovascular diseases.

### Provenance and peer review

Not commissioned, externally peer reviewed.

## Ethical approval

We have the agreement of the ethics committee, university Mohammed first faculty of medicine and pharmacy OUJDA MOROCCO.

## Sources of funding

We have any sponsors.

## Author contribution

AHMED AMINE EL OUMRI: concept. MOHAMMED NOUMAIRI, AMINE BOUALLALA, SIHAM EL MIR: collection. ALLAM ABDALLAH: analysis. MOHAMMED NOUMAIRI: writing the paper

## Consent

Wi did just review not study so we have not any consent of patients.

## Registration of research studies

Name of the registry:
Unique Identifying number or registration ID:
Hyperlink to your specific registration (must be publicly accessible and will be checked):

## Guarantor

MOHAMMED NOUMAIRI et al.

## Declaration of competing interest

- We have any conflicts of interest
- All authors agree to publication.
